# Acetylation Profiles in the Metabolic Process of Glioma-Associated Seizures

**DOI:** 10.3389/fneur.2021.713293

**Published:** 2021-10-01

**Authors:** Ya-Wen Xu, Peng Lin, Shu-Fa Zheng, Wen Huang, Zhang-Ya Lin, Huang-Cheng Shang-Guan, Yuan-Xiang Lin, Pei-Sen Yao, De-Zhi Kang

**Affiliations:** ^1^Department of Neurosurgery, Neurosurgical Research Institute, The First Affiliated Hospital, Fujian Medical University, Fuzhou, China; ^2^Department of Pain, The First Affiliated Hospital, Fujian Medical University, Fuzhou, China; ^3^Fujian Key Laboratory of Precision Medicine for Cancer, The First Affiliated Hospital, Fujian Medical University, Fuzhou, China; ^4^Key Laboratory of Radiation Biology of Fujian Higher Education Institutions, The First Affiliated Hospital, Fujian Medical University, Fuzhou, China

**Keywords:** lysine acetylation, metabolic process, glioma-associated seizures, glioma, protein-protein interaction

## Abstract

**Objective:** We test the hypothesis that lysine acetylation is involved in the metabolic process of glioma-associated seizures (GAS).

**Methods:** We used label-free mass spectrometry-based quantitative proteomics to quantify dynamic changes of protein acetylation between gliomas with seizure (CA1 group) and gliomas without seizure (CA2 group). Furthermore, differences of acetyltransferase and deacetylase expression between CA1 and CA2 groups were performed by a quantitative proteomic study. We further classified acetylated proteins into groups according to cell component, molecular function, and biological process. In addition, metabolic pathways and protein interaction networks were analyzed. Regulated acetyltransferases and acetylated profiles were validated by PRM and Western blot.

**Results:** We detected 169 downregulated lysine acetylation sites of 134 proteins and 39 upregulated lysine acetylation sites of 35 proteins in glioma with seizures based on acetylome. We detected 407 regulated proteins by proteomics, from which ACAT2 and ACAA2 were the differentially regulated enzymes in the acetylation of GAS. According to the KEGG analysis, the upregulated acetylated proteins within the PPIs were mapped to pathways involved in the TCA cycle, oxidative phosphorylation, biosynthesis of amino acids, and carbon metabolism. The downregulated acetylated proteins within the PPIs were mapped to pathways involved in fatty acid metabolism, oxidative phosphorylation, TCA cycle, and necroptosis. Regulated ACAT2 expression and acetylated profiles were validated by PRM and Western blot.

**Conclusions:** The data support the hypothesis that regulated protein acetylation is involved in the metabolic process of GAS, which may be induced by acetyl-CoA acetyltransferases.

## Introduction

Seizures are the presenting signs of patients with glioma and contribute to low quality of life (1, 2). Antiseizure medication (ASM) administration is recommended in patients with glioma-associated seizures (GAS). However, an important subset of glioma patients with seizures are refractory to ASM therapy (1). Lysine acetylation of proteins has recently been considered as a highly conserved post-translational modification (PTM) that regulates numerous enzymes of metabolic process in mitochondria, including tricarboxylic acid (TCA) cycle, oxidative phosphorylation, and metabolism of amino acids and fatty acid (3–5). Mitochondrial acetylation is generally considered as an inhibitory PTM and acetylation of specific lysine, which are known to reduce activities of multiple enzymes in the neurotransmitter transport, TCA cycle, and fatty acid oxidation (6, 7). Lysine acetylation is managed by deacetylases and acetyltransferases, which plays a key role in biological processes for signal transduction and cellular metabolism (8). Recent studies indicate that abnormal deacetylase and acetyltransferase expressions are associated with the tumorigenesis and progression of glioma (9–11). However, the role of lysine acetylation in glioma with seizures remains unknown.

Impaired oxidative and bioenergetics damage in the mitochondria has recently emerged as a critical regulator of temporal lobe epilepsy (7). In spontaneously epileptic rats, Gano et al. detected decreased expression of SIRT3 and a 60% increase of mitochondrial acetylation in chronic epilepsy (7). Seizure-induced TLR4/MYD88 signaling could be suppressed by histone deacetylase inhibitor SAHA *via* upregulated histone acetylation (12). Biomarkers of oxidative dysfunction have been observed in patients with epilepsy (13). Metabolic dysfunction is known to occur with epileptogenesis after initial brain injury (14). Dysfunction of proteins maintaining energy homeostasis frequently induces an epileptic phenotype, which indicates that energy failure contributes to epileptogenesis (14). Reduction of TCA cycle activities, including elevations of citrate and dysregulation of TCA intermediates, has been found in hippocampus of patients with epilepsy (15). Ketone supplementation has been proved to mediate seizure protection *via* inducing switch from glycolysis to fatty acid oxidation and ketone body generation (16). Significantly enhanced seizure control could be achieved by specific medium chain fatty acids compared to valproate (17). Low-dose fish oil also contributes to better seizure control compared with placebo (18). Long chain omega-3 fatty acid supplements have been shown to provide seizure reduction *via* elevation of seizure thresholds (5). Numerous bioenergetic and metabolic pathways affected by acetylation upregulation in temporal lobe epilepsy are identified by proteomics analysis (7).

The differentially acetylated proteins in glioma with seizures, as well as the relation between acetylation and metabolic process of glioma-associated epilepsy remain unclear. In the present study, we performed label-free mass spectrometry-based quantitative proteomics to quantify differentially expressed acetylation-associated proteins and dynamic changes of protein acetylation between gliomas with seizures and gliomas without seizures. We aimed to delineate affected metabolic processes by analyzing the resulting profiles of acetylated proteins *via* gene ontology (GO) and pathway analysis, especially in TCA cycle, fatty acid metabolism, and oxidative phosphorylation.

## Materials and Methods

### Tissue Collection

**Figure 1:**
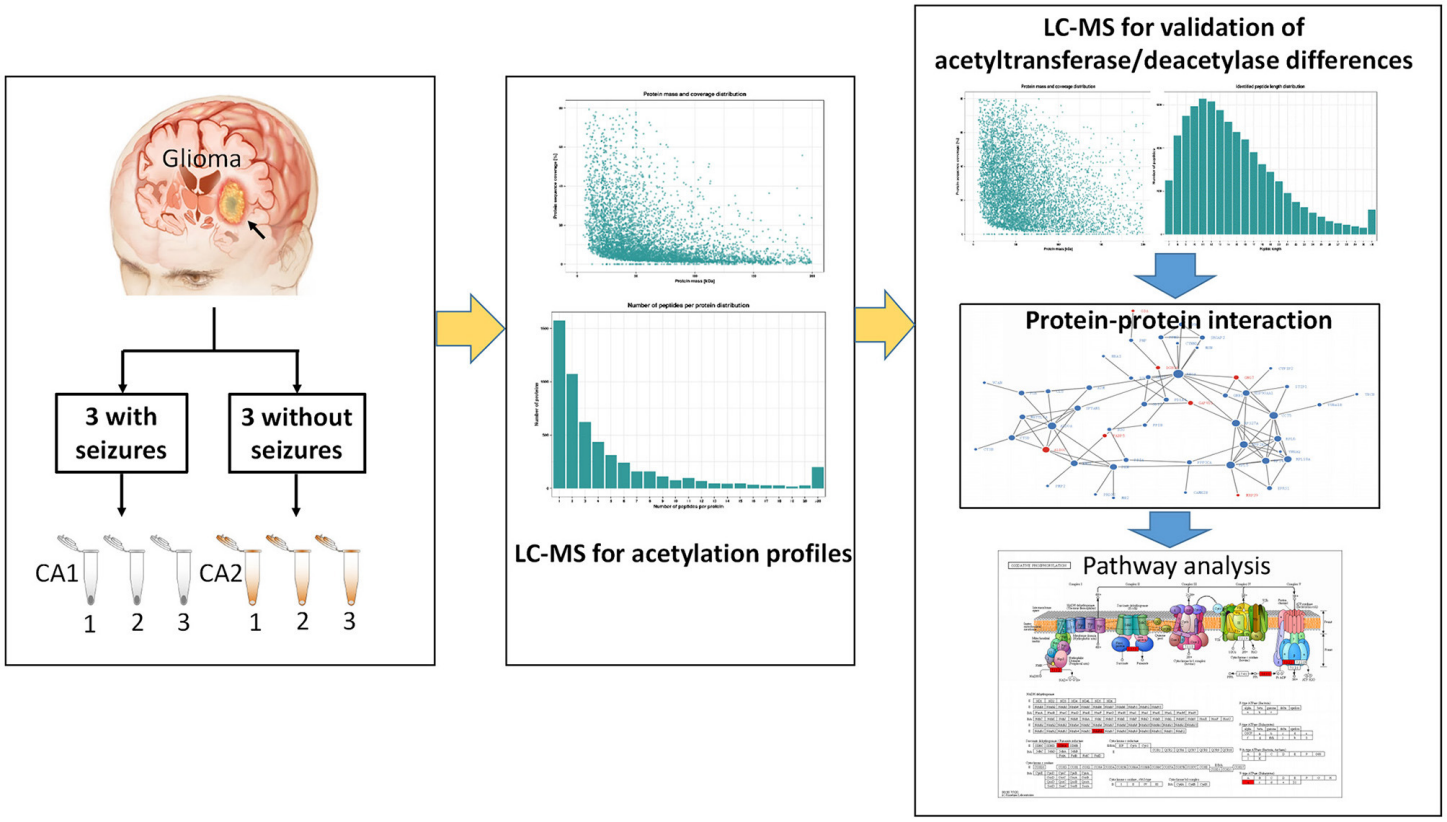
The workflow of the study.

The study protocol was approved by the ethics committee of First Affiliated Hospital of Fujian Medical University. Six gliomas (three with seizures and three without seizures) were obtained from patients undergoing resection. Three tissues surrounding gliomas were used as controls of gliomas. Samples were immediately frozen by liquid nitrogen after resection and stored at −80°C. All patients provided written informed consents. All patients underwent an electroencephalography (EEG) examination preoperatively. Seizures were confirmed by symptoms and preoperative EEG. Glioma was confirmed by postoperative histology examination. Six samples were used for label-free quantification (LFQ). Three gliomas with seizures were pooled into three samples (CA1 group) as CA1-1, CA1-2, and CA1-3, while three gliomas without seizures were pooled into three control samples (CA2 group) as CA2-1, CA2-2, and CA2-3. Figure 1 shows the workflow of this study.

### Protein Extraction and Digestion

All samples were ground into powder by liquid nitrogen and transferred to a 5-ml centrifuge tube. Subsequently, four volumes of lysis buffer (1% Protease Inhibitor Cocktail, 1% Triton X-100, 1%Phosphatase inhibitor, 3 μM TSA, 50 μM PR-619 and 50 mM NAM, and 50 mM Tris-HCl, pH 7.4) were added, followed by sonication on ice with a high-intensity ultrasonic processor (Scientz). Centrifugation (12,000 *g*) at 4°C for 10 min was performed to remove the remaining debris. Supernatant was harvested and the protein concentration was determined by BCA kit. Quality control of extracted proteins was performed by SDS-PAGE. For digestion, the protein solution was reduced by 5 mM dithiothreitol for 30 min at 37°C. Alkylation with final 11 mM iodoacetamide (IAM) was managed darkly at room temperature for 15 min. Subsequently, proteins were diluted with 200 mM TEAB. Last, trypsin was added at a ratio of 1:50 (protease:protein, m/m, PPR) for the first digestion, which was performed at 37°C overnight. Subsequently, a PPR of 1:100 for second digestion was performed for 4 h.

### Acetylation Modification Enrichment

The peptide was dissolved in IP buffer solution (100 mM NaCl, 1 mM EDTA, 50 mM Tris-HCl, and 0.5% NP-40, pH 8.0), and the supernatant was transferred to the pre-washed acetylated resin (PTM-104, PTM Biolabs, 16 mg/ml) at 4°C overnight. After incubation, the resin was washed with IP buffer solution and ionized water. The peptides bound to the resin were eluted by 0.1% trifluoroacetic acid triply. Finally, the collected peptide mixtures were desalted by C18 ZipTips and dried by vacuum centrifugation (19).

### LC-MS/MS Analysis

The tryptic peptides were dissolved in solvent A (0.1% formic acid, 2% acetonitrile). Peptides were separated in solvent B (0.1% formic acid in 98% acetonitrile) with a gradient from 7 to 22% by a reversed-phase analytical column (25-cm length, 100 μm i.d.) for 40 min and 22 to 30% for 14 min. Subsequently, solvent B was increased to 80% for 3 min and then held at 80% for 3 min. A nanoElute UHPLC system (Bruker Daltonics) was used at a constant flow rate of 350 nl/min. The peptides were subjected to Capillary source followed by the timsTOF Pro (Bruker Daltonics) mass spectrometry. The electrospray voltage was 1.7 kV. Intact peptides were detected and analyzed by TOF detector with a MS/MS scan ranging from 100 to 1,700 m/z. The data acquisition mode was performed by parallel accumulation serial fragmentation mode. Data-dependent procedures could alternate between 1 MS scan and 10 PASEF-MS/MS scans with 24.0 s of dynamic exclusion.

### Database Search

MS/MS data were processed using MaxQuant search engine (v.1.6.6.0). Homo_sapiens_9606 database (20366 entries) was searched and concatenated with reverse decoy database (contained in MaxQuant). Trypsin/P was specified as a cleavage enzyme with a maximum of two missing cleavages. The mass tolerance for precursor ions was set as 20 ppm in the first search and 20 ppm in the main search. The mass tolerance for fragment ions was set as 20 ppm. Carbamidomethyl on Cys was specified as fixed modification. Acetylation on protein N-terminal, oxidation on Met, and acetylation on Lys were specified as variable modifications. False discovery rate (FDR) was adjusted to less than 1%.

### Bioinformatics Analysis and Protein–Protein Interaction Network

The annotations on the functions and characteristics of identified proteins were performed using GO (http://www.ebi.ac.uk/GOA/). Clusters of Orthologous Groups of proteins (COG/KOG) function classification and subcellular localization were analyzed by the software wolfpsort. Pathway analysis was performed using Kyoto Encyclopedia of Genes and Genomes (KEGG) database. Further BP and KEGG enrichment were analyzed by ClueGO (v2.5.6).

All differentially expressed protein database accession or sequence were searched against the STRING database (version 10.5) for PPIs. Interactions between the proteins in the database were analyzed, including differentially acetylation-associated enzyme and acetylated proteins between two groups. Confidence score was set as more than 0.7 and disconnected nodes in the network were not shown.

### Western Blot Analysis

Equal amounts of proteins were separated by 12% SDS-PAGE and transferred onto a PVDF membrane (EMD Millipore). Subsequently, the membrane was cut into several strips to detect different target proteins according to molecular weight. Membranes were probed with primary antibodies (Anti-acetyllysine Antibody, Hangzhou, China, Cat: PTM-101, Lot: 10167J809, 1:1,000; Anti-Acetyl-CoA acetyltransferase antibody, Abcam, ab191431, 1:5,000) overnight at 4°C, followed by peroxidase-conjugated secondary antibodies (Goat anti-Mouse IgG, Thermo, Pierce, Cat: 31430, 1:10,000 dilution; Goat anti-Rabbit IgG, abcam, Cat: 97051, 1:5,000). After three washes, target proteins were detected by ECL solution (Thermo, Pierce) on FluorchemE Imaging System (ProteinSimple).

### Parallel Reaction Monitoring

During the PRM, the samples obtained were used for the validation. Protein extraction, trypsin digestion, acetylation modification enrichment, and LC-MS/MS analysis were performed as described previously (20). The resulting MS data were processed using Skyline (v.3.6). Peptide parameters were set as follows. Enzyme was Trypsin [KR/P], and max number of missed cleavages was 2. The peptide length was set as 8–25, and the variable modification was set as Carbamidomethyl on Cys and oxidation on Met. The max variable modifications were set as 3. Transition parameters were set as follows. Precursor charges were set as 2, 3; ion types were set as b, y, p; and ion charges were set as 1, 2. The product ions were from ion 3 to the last ion, and the ion match tolerance was 0.02 Da.

### Statistical Analysis

All the statistical analyses were performed using SPSS version 20.0 (IBM Corp., Armonk, NY, USA). The data are presented as the mean ± standard deviation. The significant differences of continuous data were performed by Student's *t*-test and one-way variance (ANOVA). *p* < 0.05 was deemed statistically significant.

### Data Availability

The data within the article are available. Any data not published within the article are also available in a public repository and include digital object identifiers (doi) or accession numbers to the datasets or to state that anonymized data will be shared by request from any qualified investigator.

## Results

### Mapping Lysine-Acetylated Proteins of GAS

A total of 8,518 lysine acetylation sites in 3,045 protein groups were identified, in which 4,429 sites in 1,688 proteins were quantified. A protein fold change greater than 1.5 or less than 0.67 was considered to indicate a differentially acetylated protein with a *p*-value of less than 0.05. Finally, we detected 39 upregulated lysine acetylation sites of 35 proteins and 169 downregulated lysine acetylation sites of 134 proteins (Supplementary Material 1).

### Protein Identification

#### Acetyltransferase/Deacetylase Differences Between Glioma With and Without Seizures

We used the quantitative proteomic approach to obtain a comprehensive view of the protein changes between glioma with and without seizures. We identified a total of 7,092 proteins, of which 6,114 were quantified. When the *p*-value was <0.05, a protein fold change greater than 1.5 or less than 0.67 was considered to indicate a differentially abundant protein (DAP). Finally, a total of 30 acetylation-associated enzymes were identified, of which 20 could be quantified. Acetyl-CoA acetyltransferase 2 (ACAT2, upregulated) and 3-ketoacyl-CoA thiolase (ACAA2, downregulated) were the differentially acetylation-associated enzymes in GAS. Sin3 histone deacetylase, diamine acetyltransferase 2, glucosamine 6-phosphate N-acetyltransferase, histone deacetylase 11, N-alpha-acetyltransferase 16, heparan-alpha-glucosaminide N-acetyltransferase, N-alpha-acetyltransferase 20, histone deacetylase 4, arylamine N-acetyltransferase 1, and histone deacetylase 3 could not be quantified. Upregulated ACAT2 may contribute to elevated acetylation in glioma with seizures, while downregulated ACAA2 may contribute to reduced acetylation (Table 1).

**Table 1:** Acetylation-associated enzymes obtained in three paired samples.

**Protein**	**Protein description**	**Gene name**	**CA1/CA2**	***p* value**
			**ratio**	
Q9BWD1	Acetyl-CoA acetyltransferase, cytosolic OS = *Homo sapiens* OX = 9,606 GN = ACAT2 PE = 1 SV = 2	ACAT2	2.806	0.003071136
P42765	3-ketoacyl-CoA thiolase, mitochondrial OS = *Homo sapiens* OX = 9,606 GN = ACAA2 PE = 1 SV = 2	ACAA2	0.4704	0.020749732
Q92769	Histone deacetylase 2 OS = *Homo sapiens* OX = 9,606 GN = HDAC2 PE = 1 SV = 2	HDAC2	2.1495	0.05212274
Q9BXJ9	N-alpha-acetyltransferase 15, NatA auxiliary subunit OS = *Homo sapiens* OX = 9,606 GN = NAA15 PE = 1 SV = 1	NAA15	1.6062	0.110483361
Q9UBN7	Histone deacetylase 6 OS = *Homo sapiens* OX = 9,606 GN = HDAC6 PE = 1 SV = 2	HDAC6	1.4523	0.122688072
Q9GZZ1	N-alpha-acetyltransferase 50 OS = *Homo sapiens* OX = 9,606 GN = NAA50 PE = 1 SV = 1	NAA50	1.6644	0.123019079
Q5SQI0	Alpha-tubulin N-acetyltransferase 1 OS = *Homo sapiens* OX = 9,606 GN = ATAT1 PE = 1 SV = 1	ATAT1	1.7331	0.253116561
Q5VZE5	N-alpha-acetyltransferase 35, NatC auxiliary subunit OS = *Homo sapiens* OX = 9,606 GN = NAA35 PE = 1 SV = 1	NAA35	1.2377	0.267422455
P41227	N-alpha-acetyltransferase 10 OS = *Homo sapiens* OX = 9,606 GN = NAA10 PE = 1 SV = 1	NAA10	1.3591	0.283256927
Q8WUY8	N-acetyltransferase 14 OS = *Homo sapiens* OX = 9,606 GN = NAT14 PE = 1 SV = 1	NAT14	1.7364	0.313171545
Q9Y303	N-acetylglucosamine-6-phosphate deacetylase OS = *Homo sapiens* OX = 9,606 GN = AMDHD2 PE = 1 SV = 2	AMDHD2	0.2753	0.346354931
Q8IXJ6	NAD-dependent protein deacetylase sirtuin-2 OS = *Homo sapiens* OX = 9,606 GN = SIRT2 PE = 1 SV = 2	SIRT2	0.3572	0.378204902
Q9H0A0	RNA cytidine acetyltransferase OS = *Homo sapiens* OX = 9,606 GN = NAT10 PE = 1 SV = 2	NAT10	1.2649	0.404017561
Q9NTG7	NAD-dependent protein deacetylase sirtuin-3, mitochondrial OS = *Homo sapiens* OX = 9,606 GN = SIRT3 PE = 1 SV = 2	SIRT3	1.6614	0.52583117
Q9NXA8	NAD-dependent protein deacetylase sirtuin-5, mitochondrial OS = *Homo sapiens* OX = 9,606 GN = SIRT5 PE = 1 SV = 2	SIRT5	1.2966	0.606251088
Q13547	Histone deacetylase 1 OS = *Homo sapiens* OX = 9,606 GN = HDAC1 PE = 1 SV = 1	HDAC1	1.3209	0.646583396
O00422	Histone deacetylase complex subunit SAP18 OS = *Homo sapiens* OX = 9,606 GN = SAP18 PE = 1 SV = 1	SAP18	1.3292	0.662503483
O14929	Histone acetyltransferase type B catalytic subunit OS = *Homo sapiens* OX = 9,606 GN = HAT1 PE = 1 SV = 1	HAT1	2.2573	0.674429363
P43155	Carnitine O-acetyltransferase OS = *Homo sapiens* OX = 9,606 GN = CRAT PE = 1 SV = 5	CRAT	1.0832	0.692020684
P10515	Dihydrolipoyllysine-residue acetyltransferase component of pyruvate dehydrogenase complex, mitochondrial OS = *Homo sapiens* OX = 9,606 GN = DLAT PE = 1 SV = 3	DLAT	1.1466	0.840604425
P24752	Acetyl-CoA acetyltransferase, mitochondrial OS = *Homo sapiens* OX = 9,606 GN = ACAT1 PE = 1 SV = 1	ACAT1	0.9556	0.949472071

#### Functional Annotation and Enrichment Analysis of the Regulated Acetylated Proteins

To better characterize the lysine acetylome in glioma with seizure, we performed annotation of all regulated acetylation proteins based on biological process, molecular function and cellular component, COG/KOG categories, and subcellular localization (Supplementary Materials 2–4).

**Figure 2:**
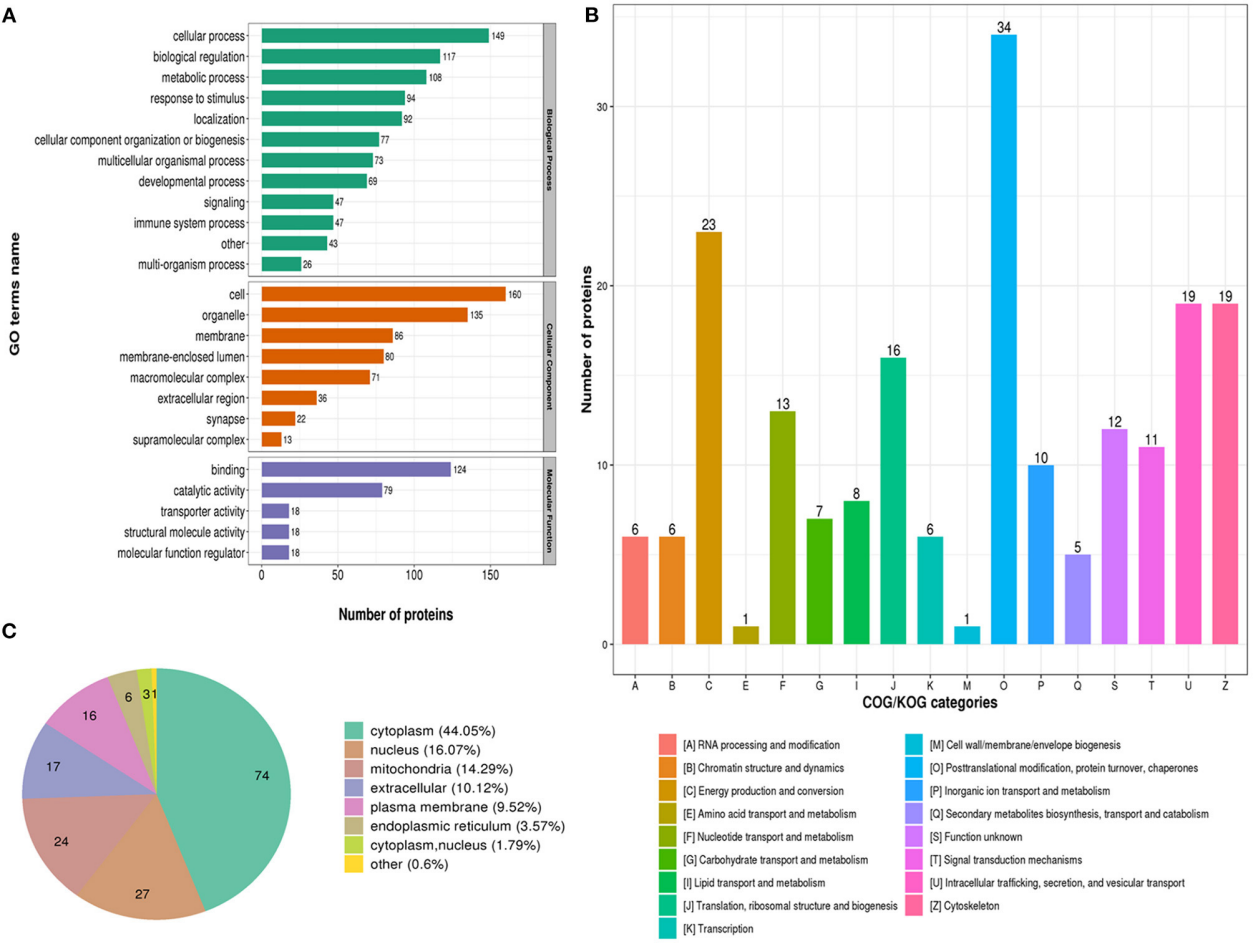
Characterization of identified regulated acetylated proteins in glioma-associated seizure. **(A)** The classification of regulated acetylated proteins in biological process, cell components, and molecular function. **(B)** The categories of regulated acetylated proteins in COG/KOG categories. **(C)** The distribution of regulated acetylated proteins in subcellular localization.

The biological process in GO analysis indicated that regulated acetylation proteins were mainly involved in the cellular process, metabolic process, and biological regulation (Figure 2A). The cell component of regulated acetylated proteins was analyzed by GO annotation. Regulated acetylated proteins distributed in various kinds of locations, mainly in cell, organelles, membrane-enclosed lumen, and membrane complex (Figure 2A). The molecular function analysis showed that of the regulated acetylated proteins, main substrates were associated with binding and catalytic activity (Figure 2A). The COG/KOG categories indicated that regulated acetylation proteins were classified as posttranslational modification, protein turnover, energy production and conversion, intracellular trafficking, secretion, and vesicular transport (Figure 2B). Subcellular localizations of the upregulated acetylation proteins were cytoplasm (44.05%), nucleus (16.07%), and mitochondria (14.29%, Figure 2C).

**Figure 3:**
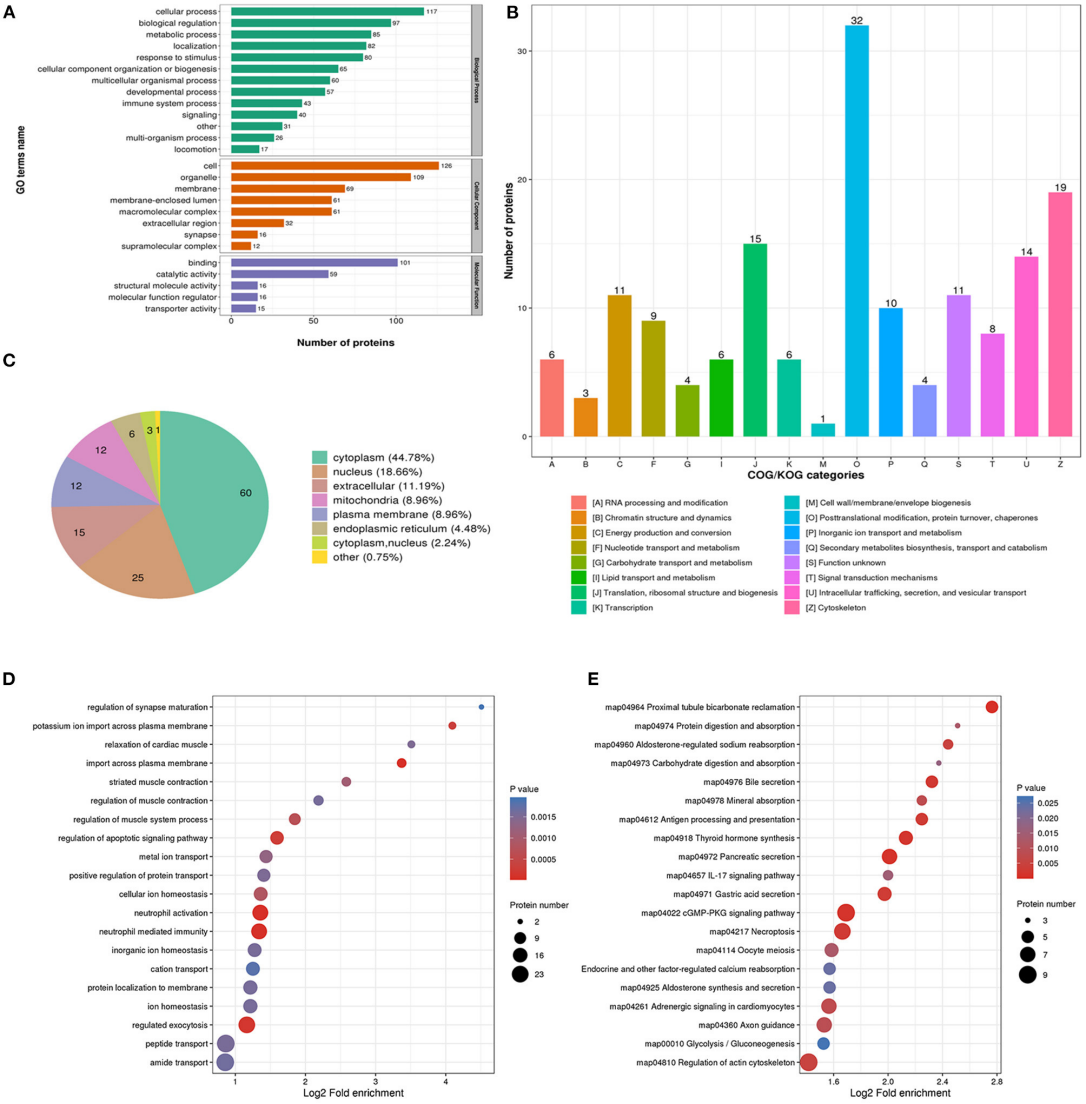
Characterization and functional enrichment analysis of identified downregulated acetylated proteins in glioma-associated seizure. **(A)** The classification of downregulated acetylated proteins in biological process, cell components, and molecular function. **(B)** The categories of downregulated acetylated proteins in COG/KOG categories. **(C)** The distribution of downregulated acetylated proteins in subcellular localization. Downregulated acetylated proteins in glioma with seizures were classified by GO annotation based on **(D)** biological process and **(E)** the KEGG pathway database.

The biological process analysis indicated that downregulated acetylation proteins were involved in the cellular process, biological regulation, and metabolic process (Figure 3A). Downregulated acetylated proteins mainly distributed in cell, membrane, and organelles (Figure 3A). The molecular function of downregulated acetylated proteins was associated with catalytic activity and binding (Figure 3A). The COG/KOG categories showed that downregulated acetylation proteins were classified as posttranslational modification, protein turnover, and cytoskeleton (Figure 3B). Subcellular localizations of the downregulated acetylation proteins were cytoplasm (44.78%), nucleus (18.66%), extracellular (11.19%), and mitochondria (8.96%, Figure 3C). Biological process of downregulated acetylated proteins in GO analysis was enriched in neutrophil activation, neutrophil-mediated immunity, regulation of apoptotic signaling pathway, and regulated exocytosis (Figure 3D, Supplementary Material 2). KEGG pathway analysis showed that downregulated acetylated proteins were involved in the pathway of fatty acid metabolism, oxidative phosphorylation, TCA cycle, metabolic pathways, and necroptosis (Figure 3E, Supplementary Material 6). Proteomics analysis indicated that ACAA2 contribute to reduced lysine acetylation in the fatty acid metabolism of GAS. Downregulated acetylated proteins in the pathway of necroptosis were SLC25A5, SLC25A6, HMGB1, HSP90AA1, HSP90AB1, PPIA, PYGB, and CAMK2B (Supplementary Material 1). Downregulated acetylated proteins in fatty acids metabolism were ACSL3, FASN, HADHB, HSD17B12, and ECI1 (Supplementary Material 1).

**Figure 4:**
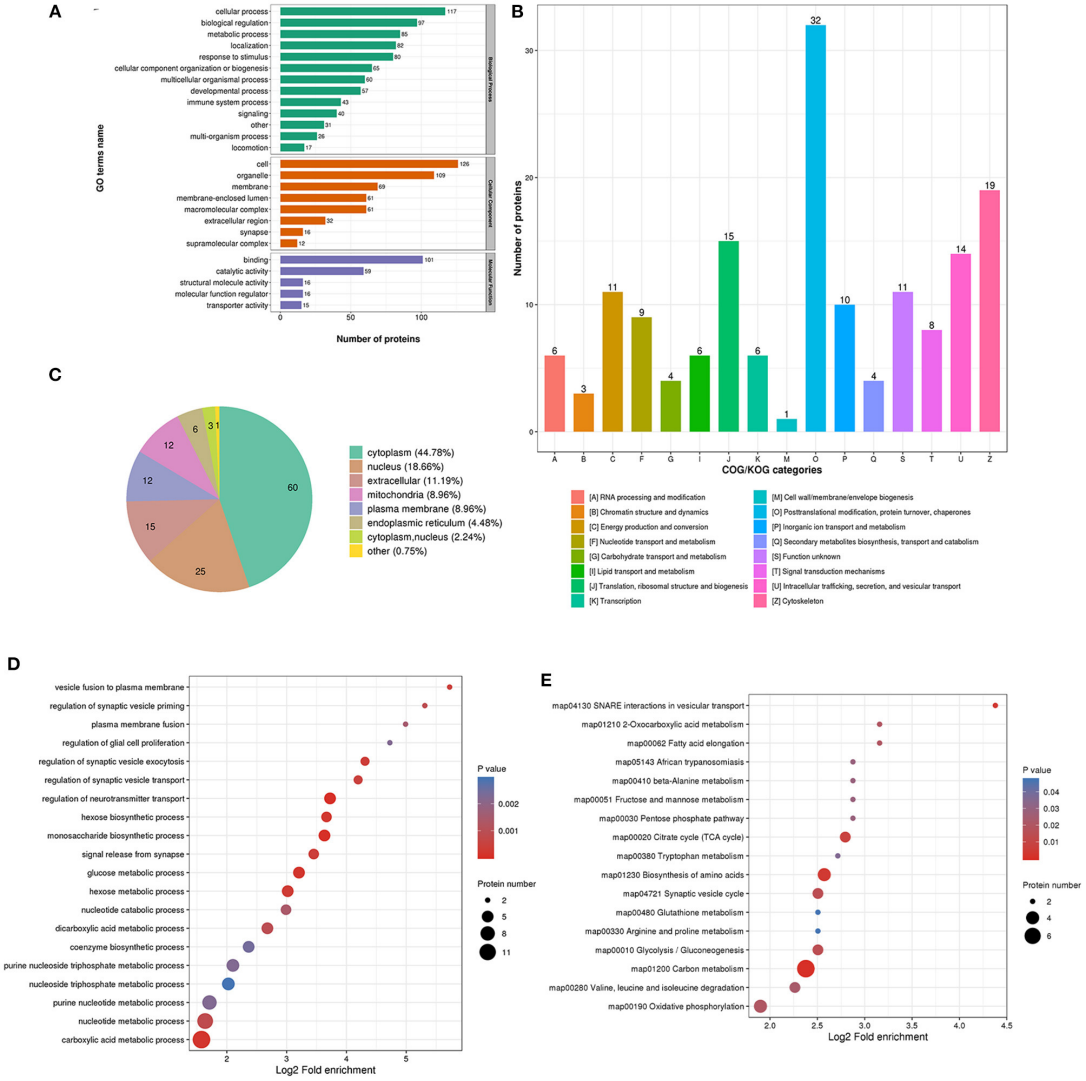
Characterization and functional enrichment analysis of identified upregulated acetylated proteins in glioma-associated seizure. **(A)** The classification of upregulated acetylated proteins in biological process, cell components, and molecular function. **(B)** The categories of upregulated acetylated proteins in COG/KOG categories. **(C)** The distribution of upregulated acetylated proteins in subcellular localization. Upregulated acetylated proteins in glioma with seizures were classified by GO annotation based on **(D)** biological process and **(E)** the KEGG pathway database.

The biological process in GO analysis indicated that upregulated acetylation proteins were mainly involved in the cellular process, metabolic process, and biological regulation (Figure 4A). The cell component of upregulated acetylated proteins was analyzed by GO annotation. Upregulated acetylated proteins distributed in various kinds of locations, mainly in cell, organelles, membrane-enclosed lumen, and macromolecular and membrane complex (Figure 4A). The molecular function analysis showed that of the upregulated acetylated proteins, main substrates were associated with binding and catalytic activity (Figure 4A). The COG/KOG categories indicated that upregulated acetylation proteins were classified as energy production and conversion, intracellular trafficking, secretion, and vesicular transport, nucleotide transport, and metabolism (Figure 4B). Subcellular localizations of the upregulated acetylation proteins were cytoplasm (42.86%) and mitochondria (34.29%, Figure 4C). To further elucidate the cellular function in glioma with seizures, we tested the data for enrichment in three GO categories: biological process, cell component, and molecular function. Biological process of upregulated acetylated in GO analysis was enriched in regulation of neurotransmitter transport, monosaccharide biosynthetic process, carboxylic acid metabolic process, glucose metabolic process, and hexose biosynthetic process (Figure 4D, Supplementary Material 2). Additionally, KEGG pathway analysis showed that upregulated acetylated proteins were enriched in the pathway of carbon metabolism, biosynthesis of amino acids, TCA cycle, glycolysis/gluconeogenesis, and oxidative phosphorylation (Figure 4E, Supplementary Material 5). The identification of acetylation-associated proteins involved in proteosome pathway hints that ACAT2 induces elevated lysine acetylation in the metabolic process of glioma with epilepsy. Upregulated acetylated proteins in the pathway of biosynthesis of amino acids were ALDOA, ALDOC, GOT2, and IDH2 (Supplementary Material 5). Upregulated acetylated proteins in the pathway of fatty acids metabolism were HADHA, ACOT7, ALDH9A1, and GOT2.

#### PPI Network

**Figure 5:**
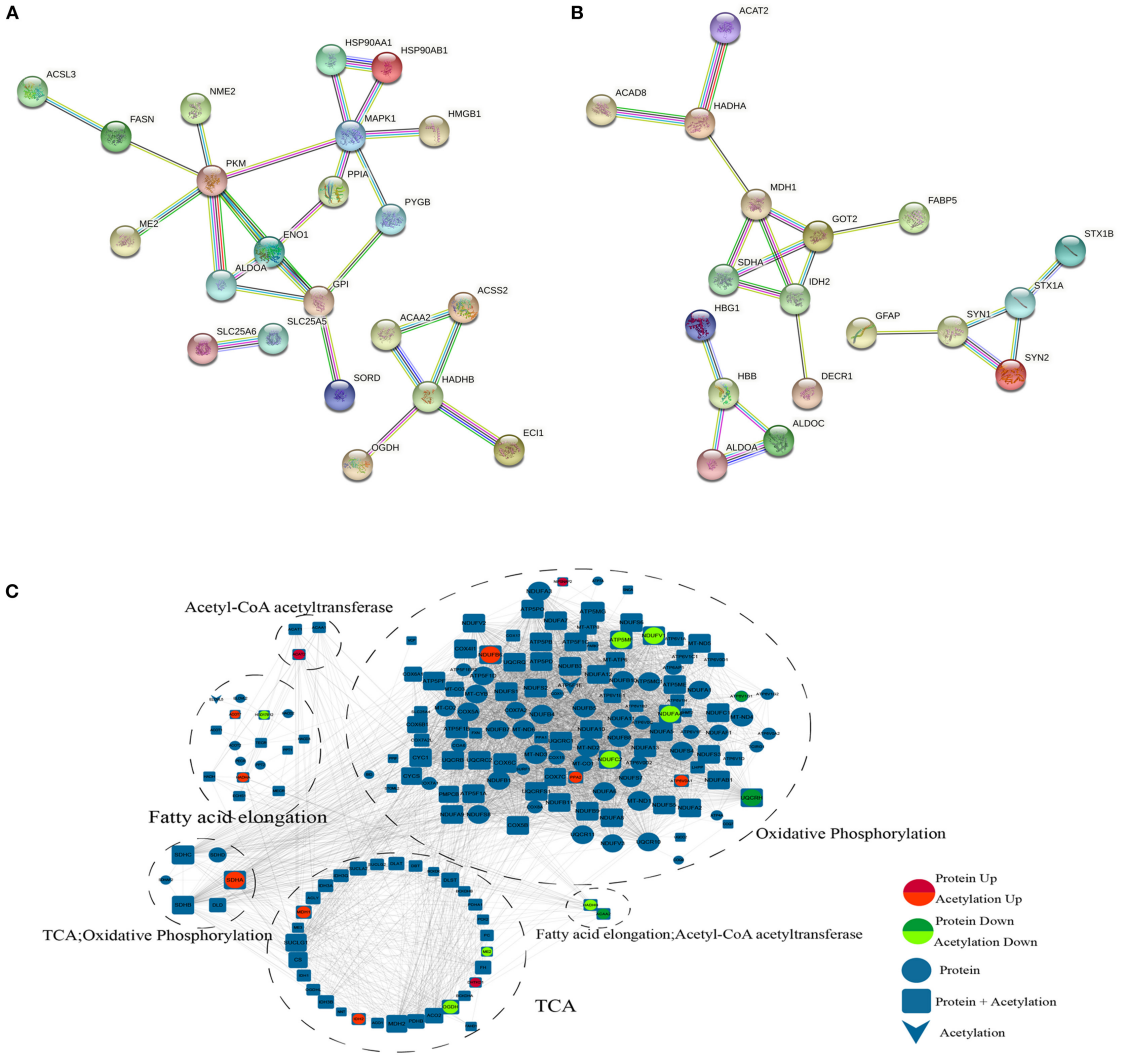
PPI network. **(A)** ACAA2 and downregulated acetylated proteins (including metabolic pathways, fatty acid metabolism, and necroptosis). **(B)** ACAT2 and upregulated acetylated proteins. **(C)** ACAA2, ACAT2, and metabolic pathway (including oxidative phosphorylation, fatty acid elongation, and TCA cycle).

According to the KEGG analysis, the downregulated acetylated proteins within the PPIs were involved in fatty acid metabolism, oxidative phosphorylation, TCA cycle, and necroptosis (Figure 5A, Supplementary Material 5). We obtained the PPI network of ACAA2 and downregulated acetylated proteins from STRING database, especially in metabolic pathways, fatty acid metabolism, and necroptosis (Figure 5A). We searched STRING database and obtained the PPI network of ACAT2 and upregulated acetylated proteins (Figure 5B). According to the KEGG analysis, the upregulated acetylated proteins within the PPIs were mainly mapped to pathways involved in the TCA cycle, oxidative phosphorylation, biosynthesis of amino acids (including glutamate and aspartate), and carbon metabolism. The proteins GOT2, ALDOA, ALDOC, and SDHA were involved in the regulation of neurotransmitter transport and monosaccharide biosynthetic process, which showed the lower *p*-value among the identified pathways (Figure 5B, Supplementary Material 5). The PPI network of ACAA2, ACAT2, and metabolic pathway (including oxidative phosphorylation, fatty acid elongation, and TCA cycle) is shown in Figure 5C.

#### Acetylated Differences Between CA1 and CA2 Groups

**Figure 6:**
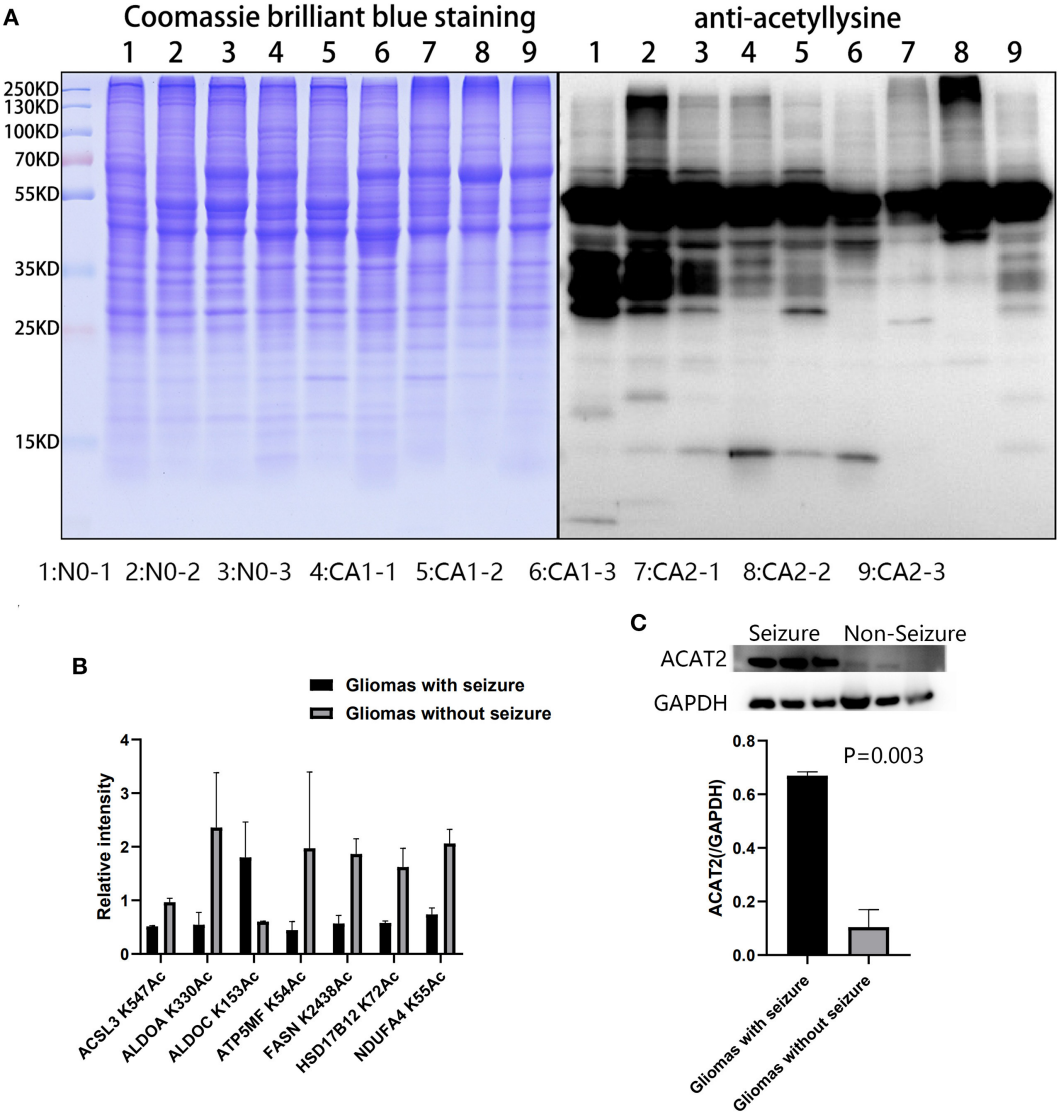
Validation of acetylated profiles and proteomics by PRM and Western blot. **(A)** Acetylation differences of brain tissues and CA1 and CA2 samples were validated by Western blot. Acetylated proteins (15–55 KD) were significantly different. Samples 1, 2, and 3 were brain tissues surrounding gliomas. Samples 4, 5, and 6 were gliomas with seizures. Samples 7, 8, and 9 were gliomas without seizures. **(B)** Seven proteins were validated by PRM, including ACSL3 K547Ac, ALDOA K330Ac, ALDOC K153Ac, ATP5MF K54Ac, FASN K2438Ac, HSD17B12 K72Ac, and NDUFA4 K55Ac. **(C)** Upregulated ACAT2 in gliomas with seizures were validated by Western blot (*p* = 0.003).

Acetylation differences between CA1 and CA2 groups were validated by Western blot (Figure 6A). Acetylated proteins (15–55 KD) were significantly different. We selected seven acetylated proteins involved in TCA cycle, oxidative phosphorylation, biosynthesis of amino acids, and fatty acid metabolism for PRM validation (Figure 6B). Regulated acetylation of seven proteins was quantified using PRM, and the results indicated that the data of acetylation profiles were reliable. Furthermore, upregulated ACAT2 in gliomas with seizures were validated by Western blot (Figure 6C, *p* = 0.003).

## Discussion

Lysine acetylation contributes to an important PTM of both histone and non-histone proteins (21). Recently, progression of glioma was found to be regulated by lysine acetylation (22, 23). Studies reported that some factors contributed to the epileptogenesis of glioma with seizure, such as tumor location, tumor type, microenvironment in peritumor, and genetic alterations (24, 25). However, the role of acetylation in glioma with seizures remains unknown. Acetyl-CoA acetyltransferases (ACATs) were known as Acetoacetyl-CoA thiolases, including mitochondrial (ACAT1) and cytosolic (ACAT2), which catalyze reversible formation of acetoacetyl-CoA from two molecules of acetyl-CoA during ketogenesis and ketolysis, respectively (26). ACAT2 plays a key role in fatty acid metabolism and cholesterol biosynthesis, which is expressed in the small intestine and liver for efficient dietary cholesterol absorption and lipoprotein assembly (27). However, ACAT2 expression and function in glioma remain unclear. The enzymatic activity of ACATs is highly activated by cholesterol through allosteric mechanism (28). ACAT2 dramatically increased following cholesterol or 25-hydroxycholesterol (25-HC) treatments in intestine and liver of mice (29). K294 acetylation in 6-phosphogluconate dehydrogenase (6-PGD) was regulated by ACAT2, which was related to NADPH production and tumor growth (30).

In the study, increased ACAT2 expression and 39 upregulated lysine acetylation sites of 35 proteins were detected in glioma with seizures. Mass spectrometry-based acetylomics data revealed a greater abundance of upregulated acetylated proteins and peptides in mitochondria and cytoplasm. Identified multiple mitochondrial pathways were regulated by protein lysine acetylation, including TCA cycle, oxidative phosphorylation, and carbon metabolism. Additionally, KEGG pathway analysis showed that upregulated acetylated proteins were enriched in the pathway of biosynthesis of amino acids, including glutamate and aspartate. The COG/KOG categories indicated that upregulated acetylation proteins were classified as energy production and conversion, intracellular trafficking, secretion, and vesicular transport, nucleotide transport, and metabolism. To our knowledge, this is the first study to show an increase in the expression of ACAT2 and increased acetylation of metabolic process-associated proteins in cerebral glioma with seizures. These results suggest a potential role for aberrant metabolic process due to protein acetylation *via* ACAT2 in glioma with seizures. Altered histone H3 and H4 acetylation has been detected in animal models of temporal lobe epilepsy (31, 32). A previous study indicated that lysine acetyltransferases 5 variants caused acetylation deficiency of histone and transcriptional dysregulation of multiples genes, which led to a neurodevelopmental syndrome with seizures (33).

In the study, ACAA2 was the unique differentially acetylation-associated enzyme, which contributes to downregulation of acetylation in GAS. ACAA2 is a member of thiolases and capable of catalyzing the Claisen condensation reaction, which contributes to achieving carbon chain elongation, production of ketone bodies, fatty acid elongation, and degradation (34). However, ACAA2 expression and function in glioma with seizures remain unclear. In the study, decreased ACAA2 expression and 169 downregulated lysine acetylation sites of 134 proteins were detected in glioma with seizures. Mass spectrometry-based acetylomics data indicated that a greater abundance of downregulated acetylated proteins were detected in cytoplasm, nucleus, extracellular, and mitochondria. The biological process analysis indicated that downregulated acetylation proteins were involved in the cellular process, biological regulation, and metabolic process. KEGG pathway analysis showed that downregulated acetylated proteins were involved in the pathway of fatty acid metabolism, oxidative phosphorylation, TCA cycle, metabolic pathways, and necroptosis. The COG/KOG categories showed that downregulated acetylation proteins were classified as posttranslational modification, protein turnover, and cytoskeleton. To our knowledge, this is the first study to show a decrease in the expression of ACAA2 and decreased acetylation of fatty acid metabolism-associated proteins in glioma with seizures. The results support the hypothesis that reduced protein acetylation is involved in the fatty acid metabolism of GAS, which may be induced by ACAA2 and served as a therapeutic target.

In the study, downregulated acetylated proteins in fatty acids metabolism were ACSL3, FASN, HADHB, HSD17B12, and ECI1. 3-Hydroxyacyl-coenzyme A dehydrogenase (HADH) catalyzes the penultimate reaction in the β-oxidation of fatty acids (35). HADH mutations were associated with epilepsy and developmental delay (36). De novo mutation of FASN in synaptic transmission genes was detected in epileptic encephalopathies *via* synaptic dysregulation (37). Carbamazepine increased the expression levels of FASN, suppressed Wnt/β-catenin expression, and was widely used in the treatment of epilepsy (38). 17-beta-hydroxysteroid dehydrogenase (HSD17B) plays a crucial role in catalyzing the androgen and estrogen biosynthesis in epileptic human hippocampus (39).

Oxidative stress and mitochondria dysfunction play a key role in the pathogenesis of epilepsy, which induce energy failure and neuronal loss in epilepsy (40). Metabolic dysfunction is known to originate from prolonged seizures and contribute to development of epilepsy (41, 42). Non-mitochondrial sources of reactive oxygen species (ROS) are also activated by seizures and involved in attenuating seizure-induced neurodegeneration (43). In spontaneously epileptic rats, decreased protein expression of SIRT3 and an increase in global mitochondrial acetylation are detected, which suggest that SIRT3 dysfunction and aberrant protein acetylation may contribute to mitochondrial dysfunction in chronic epilepsy (7). Cheng et al. reported that cortical neurons with low expression of SIRT3 exhibit abnormally elevated sensitivity to glutamate-induced calcium overload and excitotoxicity (44). Hyperacetylation of superoxide dismutase 2 and cyclophilin D in mitochondria was detected following SIRT3 deficiency, which is related to excitatory glutamatergic neurotransmission (44). Aspartate-glutamate carrier (AGC1) expressed in neurons transports aspartate from mitochondria to cytosol and plays a key role in myelination (45). Secondary hypomyelination was detected in AGC1 deficiency with lack of N-acetylaspartate (NAA), which is induced by acetylation of aspartate in neurons and essential for fatty acid synthesis (45). In the study, KEGG pathway analysis showed that upregulated acetylated proteins of glioma with seizures were enriched in the pathway of biosynthesis of amino acids, including glutamate and aspartate synthesis. Upregulated acetylated proteins in the abnormal biosynthesis of glutamate and aspartate were ALDOA, ALDOC, GOT2, and IDH2.

Ketogenesis is a metabolic process and ketone bodies are acquired from the breakdown of fatty acids (46). Acetone acts as an inhibitor of glutamate at the excitatory NMDA receptor, while it has little effect on inhibitory GABA receptors (46). Brain metabolism of ketone bodies provides as much as 30% carbon of glutamate synthesis, which possesses excitotoxicity on neurons (47). Abnormity of fatty acid metabolism was found in the study. Upregulated acetylated proteins in the pathway of fatty acids metabolism were HADHA, ACOT7, ALDH9A1, and GOT2. HADHA-related mitochondrial trifunctional protein deficiency was detected in one childhood sensory polyneuropathy with refractory seizures (48). Neurons antagonize fatty acid utilization by hydrolyzing long chain acyl-CoAs (activated form of fatty acids) *via* acyl-CoA thioesterase 7 (ACOT7) (49). Loss of ACOT7, which is a fatty acid metabolic enzyme highly enriched in neurons, contributes to increased seizure severity following kainic acid administration (49). Aldehyde dehydrogenase (ALDH) gene superfamily encodes enzymes critical for NAD(P)-dependent oxidation of aldehydes, which plays an important role in gamma-aminobutyric acid metabolism (50, 51). Mutations in ALDH genes contribute to molecular basis of several diseases, including pyridoxine-dependent seizures (50).

A review of the literature has revealed that there have been many types of PTMs of proteins (e.g., succinylation, crotonylation, 2-hydroxyisobutyrylation, malonylation, lactylation, and so on) (52), while most studies on glioma with seizure have focused on phosphorylation, which is the most common post-translational modifications of proteins. A study observed a dephosphorylation of KCC2 at residue Ser940 in a glioma model that exhibits hyperexcitability and the development of spontaneous seizures (53). Highly phosphorylated NR2B at S1013, which mediates neuronal overexcitation and seizure activity, was reported (54). A study revealed that upregulation of DAPK1 in the peritumoral tissues can phosphorylate NR2B, which could lead to glioma-induced seizures (55).

## Conclusion

Alterations of amino acid metabolism in the microenvironment of glioma plays an important role in the generation of seizures. Lysine acetylation of proteins has recently been reported to regulate metabolic process, including energy metabolism, amino acid metabolism, and fatty acid metabolism. The regulation of acetylation plays an important role on temporal lobe epilepsy. However, the role of acetylation in glioma with seizures remains unknown. We performed the acetylomics of glioma with seizures compared with glioma without seizure. The results revealed that the upregulated acetylated proteins within the PPIs were mapped to pathways involved in the TCA cycle, oxidative phosphorylation, biosynthesis of amino acids, and carbon metabolism. The downregulated acetylated proteins within the PPIs were mapped to pathways involved in fatty acid metabolism, oxidative phosphorylation, TCA cycle, and necroptosis. Regulated ACAT2 expression and acetylated profiles were validated by PRM and Western blot. ACAT2 and ACAA2 were the differentially regulated enzymes in glioma with seizure. The data support the hypothesis that regulated protein acetylation is involved in the metabolic process of GAS, including TCA cycle, oxidative phosphorylation, biosynthesis of amino acids, and fatty acid metabolism. According to the deregulated acetyltransferases, we speculated that deregulated acetylation of glioma with seizure may be induced by acetyltransferases, which might provide potential therapeutic target in controlling seizures of glioma.

## Limitations

There are several limitations as follows: First, mass spectrometry was the main method to summarize the acetylation profile of glioma-associated seizure. The corresponding antibody to the acetylation site of the relevant protein would be considered for further verification in future work. Second, this article lacked the direct evidence to verify the regulation function of acetyltransferases in the acetylation level of related proteins in glioma with seizure. Future work would be performed, which would regulate the expression or function of acetyltransferases in primary glioma cells to detect the acetylation level of related proteins. Third, the upregulation of ACAT2 was observed in glioma with seizure based on proteomics and Western blot, while the exact mechanisms that regulated the amount of ACAT2 remain unclear. Its upregulation may be caused by regulation of transcription, regulation of translational processes, or abnormalities in protein degradation. The research about regulated mechanisms may be investigated in future work. Fourth, there was lack of *in vitro* functional tests on primary isolated patient cells. This article focused on the abnormal acetylation modification of metabolism-related proteins in glioma with seizures and attempted to find potential therapeutic targets for glioma with seizures. The *in vitro* functional tests would be performed in our future work. Fifth, the sample size was small.

## Data Availability Statement

The datasets presented in this study can be found at ProteomeXchange Consortium via the PRIDE partner repository with the dataset identifier PXD027864 (https://www.ebi.ac.uk/pride/archive/projects/PXD027864).

## Ethics Statement

The studies involving human participants were reviewed and approved by First Affiliated Hospital of Fujian Medical University. The patients/participants provided their written informed consent to participate in this study.

## Author Contributions

Z-YL, Y-XL, P-SY, and D-ZK contributed to conception and design of the study. WH organized the database. H-CS-G performed the statistical analysis. Y-WX wrote the first draft of the manuscript. PL and S-FZ wrote sections of the manuscript. All authors contributed to manuscript revision, read, and approved the submitted version.

## Funding

The study was supported by Natural Science Funding of China (No. 81802492 to P-SY).

## Conflict of Interest

The authors declare that the research was conducted in the absence of any commercial or financial relationships that could be construed as a potential conflict of interest.

## Publisher's Note

All claims expressed in this article are solely those of the authors and do not necessarily represent those of their affiliated organizations, or those of the publisher, the editors and the reviewers. Any product that may be evaluated in this article, or claim that may be made by its manufacturer, is not guaranteed or endorsed by the publisher.
